# Epigenetic Silencing of IRF7 and/or IRF5 in Lung Cancer Cells Leads to Increased Sensitivity to Oncolytic Viruses

**DOI:** 10.1371/journal.pone.0028683

**Published:** 2011-12-14

**Authors:** Qunfang Li, Michael A. Tainsky

**Affiliations:** 1 Program in Molecular Biology and Genetics, Barbara Ann Karmanos Cancer Institute, Detroit, Michigan, United States of America; 2 Department of Oncology, Wayne State University School of Medicine, Detroit, Michigan, United States of America; National Cancer Center, Japan

## Abstract

Defective IFN signaling results in loss of innate immunity and sensitizes cells to enhanced cytolytic killing after Vesticular Stomatitis Virus (VSV) infection. Examination of the innate immunity status of normal human bronchial epithelial cells Beas2B and 7 lung cancer cells revealed that the abrogation of IFN signaling in cancer cells is associated with greater sensitivity to VSV infection. The disruption of the IFN pathway in lung cancer cell lines and primary tumor tissues is caused by epigenetic silencing of critical interferon responsive transcription factors IRF7 and/or IRF5. Although 5-aza-2′-deoxycytidine treatment fails to reactivate IRF7 and IRF5 expression or protect cells from VSV infection, manipulating IFN signaling by altering IRF expression changes the viral susceptibility of these cells. Lung cancer cells can be partially protected from viral killing using IRF5+IRF7 overexpression, whereas IFN pathway disruption by transfection of siRNAs to IRF5+IRF7 increases cells' vulnerability to viral infection. Therefore, IRF5 and IRF7 are key transcription factors in IFN pathway that determine viral sensitivity of lung cancer cells; the epigenetically impaired IFN pathway in lung cancer tissues provides potential biomarkers for successful selective killing of cancer cells by oncolytic viral therapy.

## Introduction

As the leading cause of cancer-related mortality in both men and women, lung cancer is responsible for well over 1 million deaths worldwide annually. Although diagnosis and treatment have been improved, the five-year survival rate is only 14% largely due to the failure of tumor debulking surgery and systemic chemotherapy. The improvement of lung cancer treatment is a major public health goal. Recently, naturally occurring or genetically engineered oncolytic viruses, including measles virus, Newcastle Disease Virus (NDV), VSV, adenoviruses, reovirus and Herpes simplex virus offer an effective and promising alternative therapeutic approach to fight this disease [1]. Used alone or in combination with chemotherapy, oncolytic viruses selectively destroy tumor cells by targeting cancer defects in major pathways, such as p53 tumor suppressor, ras signal transduction and IFN signaling pathways [1],[2]. Currently the effectiveness and safety of different oncolytic viruses in treatment of various cancers is being evaluated in preclinical animal models and phase I–III clinical trials [3]. Among them, a negative strand RNA virus VSV, which can trigger innate immunity mechanisms, has been shown to be efficacious against malignant glioma, melanoma, leukemias, hepatocellular, breast, bladder and prostate cancers that have defective antiviral responses. [4], [5], [6], [7].

Type I IFN signaling pathway is activated by VSV infection as first line innate immune response to protect normal tissues from viral killing, and therefore tumor cells that have lost their antiviral reactivity represent selective targets for VSV. The primary response upon viral infection and uptake of double-stranded RNAs is TLR3 activation which is mediated by IRF-3, cJUN/ATF-2, and NFκB, thereby inducing the production of immediate-early response genes primarily IFNβ. Those early response IFNs bind to type I IFN receptors (IFNAR) in an autocrine or paracrine manner to activate STAT1 and induce expression of secondary antiviral response genes including the transcription factor IRF7 which then promotes the expression other IFN stimulated genes (ISGs). Finally, the tertiary transcriptional wave of IFNα establishes an antiviral state [8], [9].

The impairment of IFN signaling is linked to an enhanced risk of tumor development [10], [11], [12] as the IFN pathway also exhibits antiproliferative and immune surveillance activities against cancer. Accordingly, the majority (∼80%) of NCI 60 panel cancer cell lines display disrupted innate immunity responses [9]. We have shown that the IFN signaling pathway was abrogated during spontaneous immortalization in fibroblasts from Li-Fraumeni Syndrome (LFS) patients, who are predisposed to early onset and multiple tumors because of germ-line mutations in p53. As an important epigenetic control mechanism, DNA hypermethylation of CpGs in promoter regions represses gene expression both during development and tumorigenesis. Several ISGs were down-regulated by epigenetic silencing during immortalization, an early and necessary step in carcinogenesis, and some of the same ISGs were up-regulated upon replicative senescence [13], [14], [15]. Treatment of the immortal LFS cell lines with 5-aza-2′-deoxycytidine (5-aza-dC), an inhibitor of DNA methyltransferases restored IFN signaling and induced a senescence-like state [13], [15].

The IFN-inducible transcription factors, IRFs, are essential mediators of the IFN-response. Lack of *IRF7* expression corresponded to aberrant promoter hypermethylation of CpG islands within its promoter and was also identified as one of methylation-silenced genes in several cancer types including lung, hepatocellular, gastric and pancreatic cancers [16], [17], [18], [19]. Reduced expression of IRF5, another important transcription factor of the IFN pathway, was also observed in hematological malignancies, which is consistent with its role to induce G2-M growth arrest and apoptosis [20]. Epigenetic inactivation of *IRF5* was similarly observed in hepatocellular and gastric cancer [21], [22]. As direct inducers of IFN pathway, IRF7 and IRF5 induce overlapping ISG transcriptional profiles, however, differential expression patterns and kinetics of ISGs indicted that they possess nonredundant and distinct roles in innate immune responses. Compared to IRF7, IRF5 is a much stronger activator of the early antiviral genes including IFNβ [23]. In a recent report we demonstrated that enhanced expression of IRF5 and/or IRF7 could reactivate IFN related genes, inhibit cell growth, and induce senescence [15]. Silencing of these essential IRFs and the growth-suppressive IFN pathway may be a necessary early event in the development of cancer, particularly associated with immortalization.

Although cancer cells, with their IFN-pathway-abrogated, may have acquired a growth/survival advantage over their normal counterparts, they simultaneously compromise their antiviral protective ability. Here, new therapeutic paradigms involving oncolytic RNA viruses to target the defective innate immune system in cancer cells are being explored. We found that the sensitivity to oncolytic VSV was strongly and significantly associated with the disruption of the IFN signaling pathway. A failure to up-regulate ISG expression upon dsRNA stimulation indicated a weakened antiviral response sensitizing lung cancer cell lines to VSV-induced oncolytic cell death. However, not all lung cancer cells can be killed by VSV infection, as some of them possess a relatively normal innate immunity pathway and therefore are resistant to VSV-mediated viral killing. Bisulfite sequencing revealed promoter hypermethylation of either *IRF7* and/or *IRF5* in several lung cancer cell lines. Similarly, when we investigated DNA from fresh frozen lung cancer tissues, we observed promoter methylation of *IRF7* and/or *IRF5*, suggesting that their IFN antiviral response was also epigenetically silenced as functional IRF7 and IRF5 are required for an intact IFN pathway. However 5-aza-dC treatment failed to reactivate IRF5 or IRF7 expression or rescue lung cancer cells from VSV infection. Altering innate immunity by manipulating IRF expression changed viral susceptibility of normal Beas2B bronchial epithelial cells or lung cancer cells. Cells without a functional IFN response are partially protected from virus following IRF5 and IRF7 overexpression, whereas disruption of IFN signaling by targeting IRF5 and IRF7 using siRNAs increased Beas2B cells' vulnerability to the cytolytic effects of VSV. Therefore, IRF5 and IRF7 are pivotal factors in IFN pathway that determine the viral sensitivity of the cells to oncolytic viruses. The highly selective VSV killing of lung cancer cells with an impaired IFN pathway due to epigenetically downregulation of IRFs indicates that these genes are ideal biomarkers for determining the susceptibility of tumors to oncolytic viral therapy.

## Results

### IFN signaling deficiency is associated with VSV sensitivity

The IFN pathway controls the cellular response to viral infection and dsRNA. Cells that have a fully functional innate antiviral system are able to protect themselves against viruses, largely due to the induction of IFN signaling cascade. We have shown that the IFN pathway was epigenetically inactivated in fibroblasts from LFS patients after spontaneous immortalization [14]. Examination of innate immunity status in normal bronchial epithelial cell line Beas2B, its *ras* transformed derivative cells and 2 tumorigenic clones revealed that as IFN signaling activity decreased the sensitivity to VSV killing increased during lung tumorigenesis (Li and Tainsky, unpublished data). Because functional inactivation of IFN pathway has been a common trait of many cancers, we used 7 long-term lung cancer cell lines (4 adenocarcinomas: CRL5800, CRL5807, CRL5810 and CRL5872, 2 squamous carcinomas: HTB172 and CRL5928 and 1 small cell carcinoma: CRL5869) to study the changes in their innate immune system.

Using a representative set of ISGs in Q-RT-PCR assays, we examined both the baseline ISG expression levels and their expression after stimulation of the IFN pathway by synthetic dsRNA polyI:C, which mimics viral RNA [15]. We found lower baseline expression of most ISGs tested in all lung cancer cell lines as compared to Beas2B cells (Table 1). The IFN pathway could be activated in Beas2B cells as 12 out of 14 genes were inducible by polyI:C stimulation. In contrast, polyI:C failed to induce mRNAs of all 14 genes tested in CRL5810 cells, while variable induction deficiencies of essential antiviral response genes such as *IFNα*, *IFNβ*, *IRF7*, *IRF5* and *STAT1* were detected in CRL5800, CRL5807, CRL5872 and CRL5869 cells. Surprisingly, Q-RT-PCR demonstrated polyI:C inducible expression of 10 out of 14 ISGs in HTB182 and CRL5928 cells at similar levels to Beas2B indicating a relatively normal antiviral response in those two lung cancer cells (Table 2).

**Table 1:** Basal levels of selected ISGs were down-regulated in human lung cancer cell lines compared to Beas2B cells.

	CRL5800	CRL5807	CRL5810	CRL5872	HTB182	CRL5928	CRL5869
**TLR3**	−36.00	−31.34	−23.43	−21.41	−2.89	−1.39	1.47
**IFNα**	−7.01	−7.46	−4.53	−5.89	−5.54	−1.17	4.06
**IFNβ**	−35.75	−19.03	−15.35	−4.11	−5.90	1.67	1.11
**IRF5**	−1.87	−8.46	−3.86	−2.57	−1.01	−2.03	1.80
**IRF7**	−22.32	−6.73	−25.99	−66.26	−4.53	−13.36	−4.79
**IRF8**	2.27	−1.02	−1.65	4.86	−1.69	30.27	47.18
**Stat1**	−12.55	−15.24	−6.54	−32.45	−1.87	−2.25	−2.38
**OAS1**	−19.16	−9.06	−92.41	7.94	−4.53	−1.96	2.01
**IFI15**	−143.01	−20.97	−519.15	−270.60	−28.64	−24.76	−77.71
**IFI78**	−109.90	−7.57	−232.32	−12.38	1.60	−16.91	−2.60
**IFNAR1**	−4.35	−2.91	1.20	−7.11	−1.66	−6.15	−1.10
**IRF3**	−13.36	−20.97	−36.76	−7.31	−21.26	−7.21	1.44
**IFI16**	−112.99	−2.95	−308.69	−55.33	−52.71	−172.45	−9.45
**IFI27**	−26.91	−2592.27	−436.55	−6.36	−689.78	−2.43	−27.86

**Table 2:** Abrogation of IFN pathway activation in human lung cancer cell lines after polyI:C treatment.

	Beas2B T/U	CRL5800 T/U	CRL5807 T/U	CRL5810 T/U	CRL5872 T/U	HTB182 T/U	CRL5928 T/U	CRL5869 T/U
**TLR3**	1.22	3.46	2.41	1.27	1.04	2.85	2.22	−1.27
**IFNα**	3.54	1.89	2.44	−2.46	−1.77	−1.17	2.27	−1.91
**IFNβ**	25.58	2.30	1.69	−3.46	−1.73	1.71	1.26	1.40
**IRF5**	2.15	−3.03	1.84	−3.01	−2.41	2.77	1.23	−1.44
**IRF7**	13.96	1.03	1.33	1.79	1.49	7.31	8.17	−1.30
**IRF8**	9.45	1.84	8.34	−1.80	−1.80	−1.16	1.08	−2.22
**STAT1**	14.25	1.93	1.54	2.16	1.42	10.56	5.62	−3.05
**OAS1**	15.85	2.81	6.32	−1.95	−2.19	7.41	12.82	−1.41
**IFI15**	27.28	2.53	5.74	−1.80	1.64	39.12	75.06	−1.45
**IFI78**	28.76	−1.34	5.54	−2.64	−2.03	6.50	48.50	−2.10
**IFNAR1**	1.34	−1.64	−1.40	1.18	−1.39	1.57	1.24	−9.25
**IRF3**	4.95	2.23	−1.42	1.72	−1.62	6.15	3.73	−9.13
**IFI16**	35.62	1.40	−1.38	−2.33	−1.04	24.59	17.88	−5.24
**IFI27**	566.7	133.44	121.10	−16.45	−1.33	675.59	8.63	−55.33

T/U: treated with polyI:C versus untreated cells.

**Figure 1:**
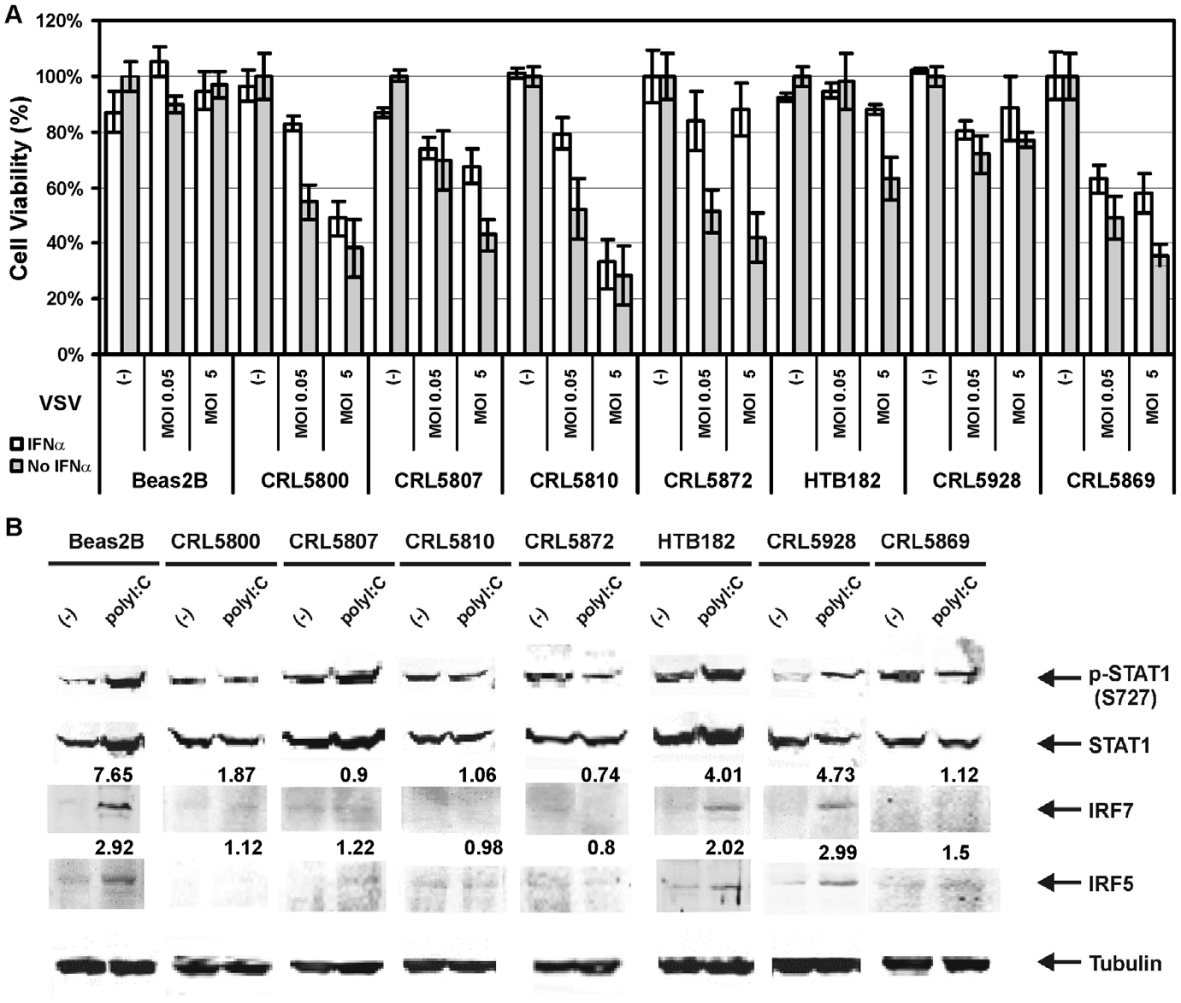
VSV sensitivity was directly correlated with the status of the IFN pathway of the cells. A. Selective cytotoxicity of VSV in lung cancer cells with defective IFN pathway. Beas2B and 7 lung cancer cell lines were evaluated for their ability to induce antiviral response upon VSV infection with or without IFNα pretreatment by virus-induced cytopathicity using MTT assay. The values were normalized to the value of control uninfected cells, which was set to 100% from at least two independent experiments (*n* = 3) with SD at <10%. (-): No VSV infection control. MOI0.05: multiplicity of infection 0.05, low dose of VSV infection. MOI5: multiplicity of infection 5, high dose of VSV infection. B. Protein expression levels of several ISGs in polyI:C treated Beas2B and lung cancer cells were compared to untreated cells using western blots. Fold changes of IRF5 and IRF7 expression after polyI:C treatment were indicated. Tubulin was used as a normalizing control.

Because compromised innate immune signaling often leads to increased sensitivity to viral killing, VSV sensitivity was investigated to determine whether the lack of ISG activation corresponds to elevated vulnerability to oncolytic viral killing in the lung cancer cells. As expected, Beas2B cells with intact IFN response resisted VSV infection exhibiting little change in cell viability. In addition, HTB182 and CRL5928 were relatively resistant compared to other lung cancer cells as more than 60% of the cells were still alive at high dose VSV (MOI5) even without exogenous IFNα pretreatment (Figure 1A). In contrast, the rest of the lung cancer cell lines variably lost their ability to protect themselves from VSV. An increasing number of IFN-signaling-deficient lung cancer cells (from ∼30% to ∼70%) were killed by raising dose of VSV exposure and adding IFNα to the medium failed to inhibit cytotoxicity of high dose VSV (MOI5) in CRL5800, CRL5810 and CRL5869 cells (Figure 1A). Interestingly, the reduced basal levels of most ISGs in all lung cancer cell lines suggested no association between VSV susceptibility and basal ISG levels (Table 1). The variable sensitivity to viral killing corresponded to the differential abrogation of the IFN response in lung cancer cell lines. The selective virolytic effects of VSV were most significant in CRL5810 cells consistent with the most severe defects in their innate anti-viral system (Table 2 and Figure 1A). We also identified 5 ISGs (*IRF5*, *IRF7*, *STAT1*, *IRF3* and *IFI16*), whose expression was distinctively elevated >3-fold in response to poly I:C treatment only in VSV-resistant cell lines (Table 2 and Figure 1A). Western blot analysis confirmed that the elevated ISG mRNA expression upon polyI:C induction resulted in upregulated protein levels in VSV-resistant cells. Ser727 phosphorylation of STAT1 can only be induced by IFNβ but not by IFNα [24], and was used as a specific marker for early response gene activation of IFN pathway. A strong polyI:C-induction of phosphorylated-STAT1 (p-STAT1, Ser727), STAT1, IRF5 and IRF7 protein levels was consistent with the resistance of normal Beas2B cells and the two cancer cells, HTB182 and CRL5928, to VSV infection and vice versa for the viral-sensitive lung cancer cell line. Mild Ser727 phosphorylation of STAT1 can be detected in CRL5807 cells indicating relatively normal early IFN signaling in this cell line compared to other VSV-sensitive cell lines. IRF5 and IRF7 protein levels were uniformly not inducible upon polyI:C treatment in all the virus-sensitive lung cancer cell lines (Figure 1B). No significant change of IRF3 protein was observed by polyI:C treatment among all lung cancer cells perhaps because its activity is mainly regulated post-translationally by changes in phosphorylation (data not shown). Therefore, our observations supported the inverse association between the oncolytic sensitivity to VSV and the inducibility of IFN signaling in normal bronchial epithelial cells Beas2B and lung cancer cells.

### Epigenetic silencing of critical transcription factors *IRF7* and *IRF5* results in abrogation of IFN pathway

**Figure 2:**
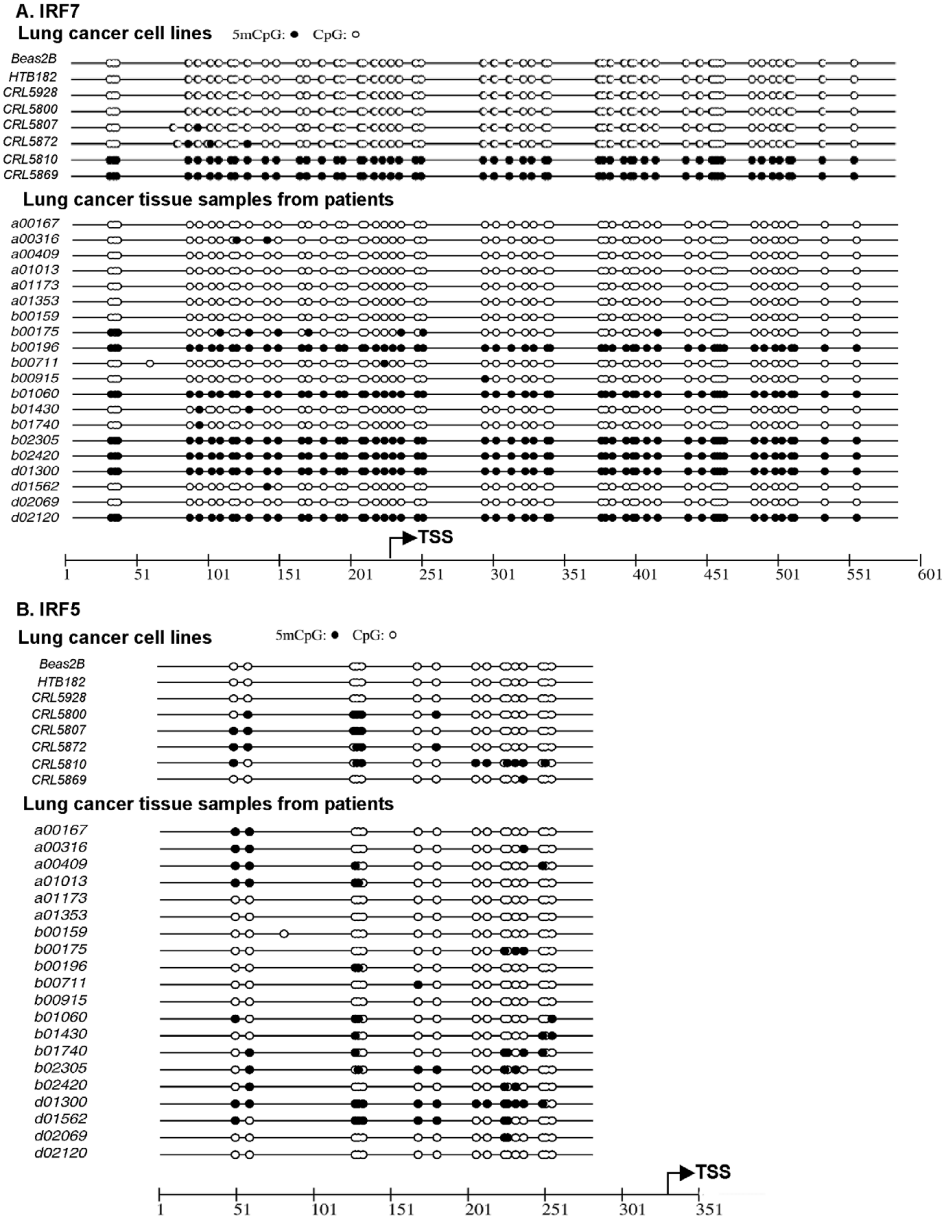
Sequencing of bisulfite treated genomic DNA revealed promoter methylation of *IRF7* and *IRF5* in lung cancer cell lines and primary tissues. The methylation status of CpG islands in the *IRF7* and *IRF5* promoter regions in Beas2B, human lung cancer cell lines and primary tissues was examined by sequencing of bisulfite treated genomic DNA. A. IRF7. B. IRF5. Closed circles, methylated C in CpG dinucleotides; open circles, unmethylated C in CpG dinucleotides. TSS, transcription start site.

Promoter hypermethylation is an epigenetic mechanism of gene regulation known to silence gene expression in mechanisms of cell fate determination and carcinogenesis. We previously reported that the IFN pathway has been abrogated by epigenetic silencing of a key antiviral defense mediator IRF7 in immortal LFS fibroblasts [14], [15]. Interestingly, another study found cigarette smoke exposure led to suppression of IFN signaling due to *IRF7* promoter hypermethylation, which resulted in decreased antiviral defenses of the respiratory epithelium [25]. Therefore, we investigated whether promoter methylation of *IRF7* could also be the cause of IFN pathway disruption in lung cancer cell lines. DNA sequencing of sodium bisulfite-treated genomic DNA revealed *IRF7* promoter hypermethylation in 2 lung cancer cell lines (CRL5810 and CRL5869), which suggests that epigenetic silencing of *IRF7* has played a role in the disruption of IFN signaling in these cell lines (Figure 2A). Since IRF5 induced a stronger transcription profile of the early antiviral genes [23] and has been newly identified as a novel methylation marker for cancer [21], [22], we further examined the methylation status of *IRF5* promoter regions in lung cancer cells. Increasing *IRF5* hypermethylation was found in CRL5800, CRL5807, CRL5872 and CRL5810 cell lines (Figure 2B), thus the similar virus susceptibility of *IRF7*-unmethylated CRL5800, CRL5807, CRL5872 cells was the consequence of epigenetic *IRF5* inactivation. Moreover, promoter hypermethylation of both *IRF7* and *IRF5* explained the highest sensitivity to VSV manifested by CRL5810 cells. In contrast, promoter regions of neither *IRF7* nor *IRF5* were found to be methylated in Beas2B, CRL5928 and HTB182 cell lines consistent with their intact innate immunity and resistance to oncolytic viral killing. Altogether the selective loss of viral protection in lung cancer cells was related to epigenetic inactivation of at least one of the IRFs implicating the necessity of both IRF7 and IRF5 to be active for a functional IFN pathway.

Because epigenetic events may occur during long-term culture, which were not present in the original cancer, we examined IRF-promoter methylation patterns in fresh-frozen primary lung cancer tissues. The *IRF7* promoter was fully methylated in 6 out of 20 NSCLCs, while 5 other tumors had 5′-partial methylation (Figure 2B) as an initial event that can spread to neighboring CpG sites [26]. In parallel, we found heavy methylation in 4 of 20 NSCLC samples and 11 others had partial methylation of the *IRF5* promoter regions. Overall, 15 out of 20 patient samples had promoter methylation of either one or both IRFs, events sufficient to attenuate their IFN response. No aberrant *IRF7* or *IRF5* hypermethylation was detected in the matching buffy coat DNAs from these same patients indicating that the IRF promoter hypermethylation had not resulted from germ-line epigenetic changes. Therefore the methylation *IRF7* or *IRF5* promoters found in the lung cancer cell lines probably had its origin in the tumor rather than being an event selected during to cell culture. Our results demonstrated that the increased susceptibility to viral infection is mediated by epigenetic mechanisms down-regulating key antiviral defense pathway regulators IRF5 and IRF7. The prevalence of epigenetic silencing of IRFs and its tight association with VSV sensitivity make them ideal theranostic biomarkers to screen lung cancer patients for possible oncolytic viral therapy.

### Manipulation of IFN signaling pathway targeting IRFs alters VSV viral sensitivity

**Figure 3:**
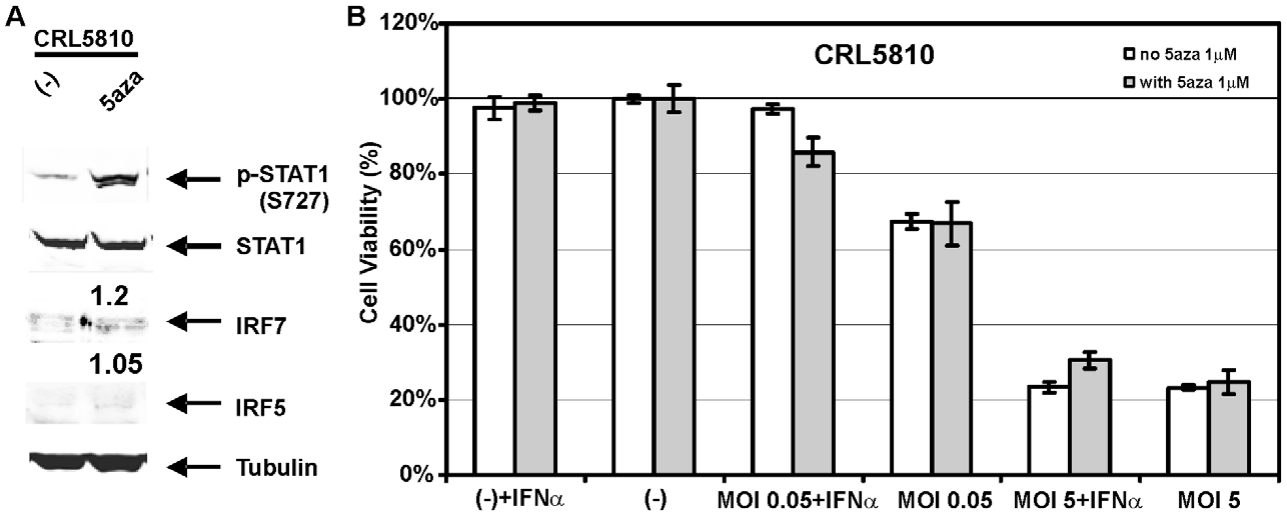
5-aza-dC treatment failed to reactivate IFN pathway or protect CRL5810 cells from VSV infection. A. Western blots revealed no increase of IRF7 or IRF5 protein expression after 5-aza-dC treatment of CRL5810 cells. Fold changes of IRF5 and IRF7 expression were indicated on top of the 5-aza-dC treated column. B. CRL5810 cells were still sensitive to VSV cytolyic effects after 5-aza-dC treatment.

We previously demonstrated that 5-aza-dC demethylation treatment could restore gene expression of epigenetically silenced *IRF7* and other ISGs thereby reactivating the IFN signaling pathway in immortal LFS fibroblast cell lines [14], [15]. To our surprise, IRF7 and IRF5 expression levels remained absent (Figure 3A, less than 1.5 fold increase for both IRF5 and IRF7) in lung cancer cell line CRL5810 after 5-aza-dC treatment for as long as 4 weeks. Remarkably, sequencing of bisulfite-treated DNA at the *IRF5* and *IRF7* promoter regions revealed that prolonged 5-aza-dC application was not able to reverse promoter hypermethylation of IRFs in CRL5810 cells (data not shown). Similar resistance to 5-aza-dC treatment was found for the other IRF7-methylated lung cancer cell line CRL5869 (data not shown). As a result, extended demethylation treatment with 5aza-dC was unable to affect the VSV sensitivity of CRL5810 cells (Figure 3B) and further indicate that IRF5 and IRF7 are two of the fundamental factors in IFN signaling that can regulate oncolytic viral sensitivity.

**Figure 4:**
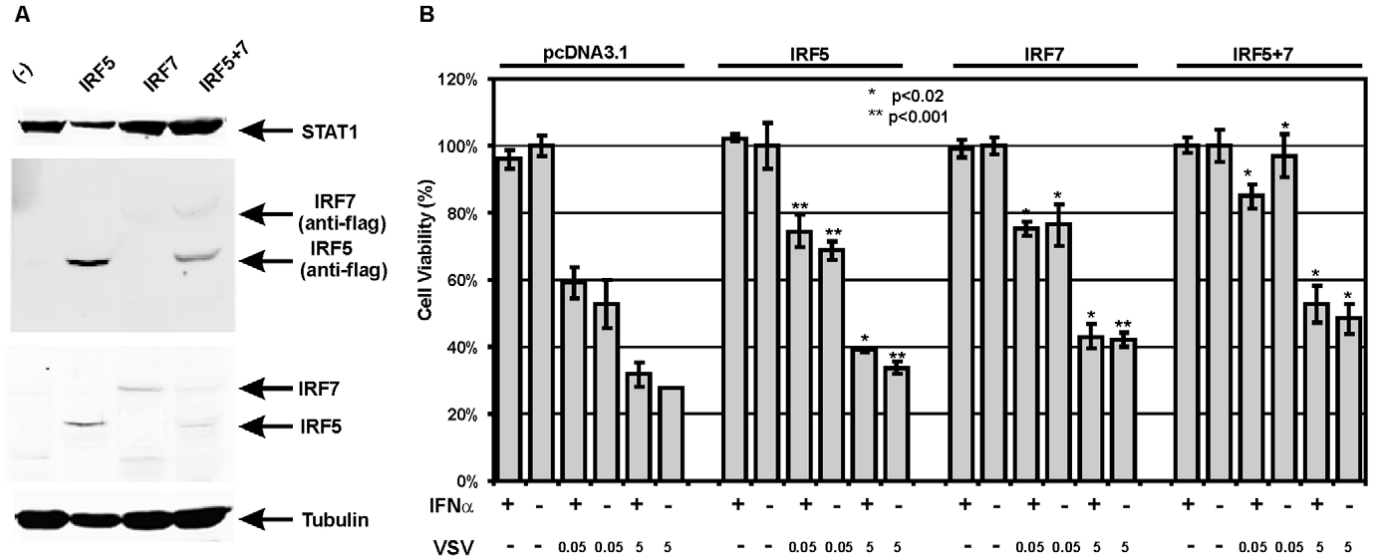
Overexpression of IRF5 and/or IRF7 partially rescued CRL5810 cells from cytolytic effect of VSV. A. Protein expression levels of STAT1, IRF5 and IRF7 were analyzed by western blots in IRF stable transfected CRL5810 cells. Anti-flag antibody was applied to detect transfected IRF protein levels, while IRF5- and IRF7-specific antibodies were used for total IRF protein expression. B. Overexpression of IRF5 and/or IRF7 partially increased CRL5810 cells' resistance to VSV infection with the most increase in the combined trasfection. * P<0.02, ** p<0.001.

Neither of these two IRF transcription factors was able to be induced in VSV-sensitive lung cancer cells (Table 2 and Figure 1B), whereas overexpression of IRF5 and/or IRF7 in immortal LFS fibroblasts upregulated other ISGs, manifested a faster and stronger innate immune signaling upon dsRNA stimulation, which is sufficient to induce senescence [15]. In order to revive the disabled IFN response in lung cancer cells, IRF5 and IRF7 alone or in combination were stably transfected into CRL5810 cells, in which sustained 5-aza-dC treatment had no effect on demethylation of IRF promoter. Western blot analysis confirmed the elevated basal protein expression of IRFs in IRF5 and IRF7 overexpressing cells (Figure 4A). Individual restoration of IRF expression partially protected CRL5810 cells from VSV cytolysis with the greatest increase of cell viability from ∼50% to more than 85% at MOI0.05, and from ∼30% to ∼50% at MOI5 of VSV infection in cells overexpressing both IRF5 and IRF7 compared to vector control cells (Figure 4B). We explained the modest gain of viral protection even after IRF5 and IRF7 combined transfection by severe loss of other important ISGs in CRL5810 cells as indicated by the lack of effect upon exogenous IFN.

**Figure 5:**
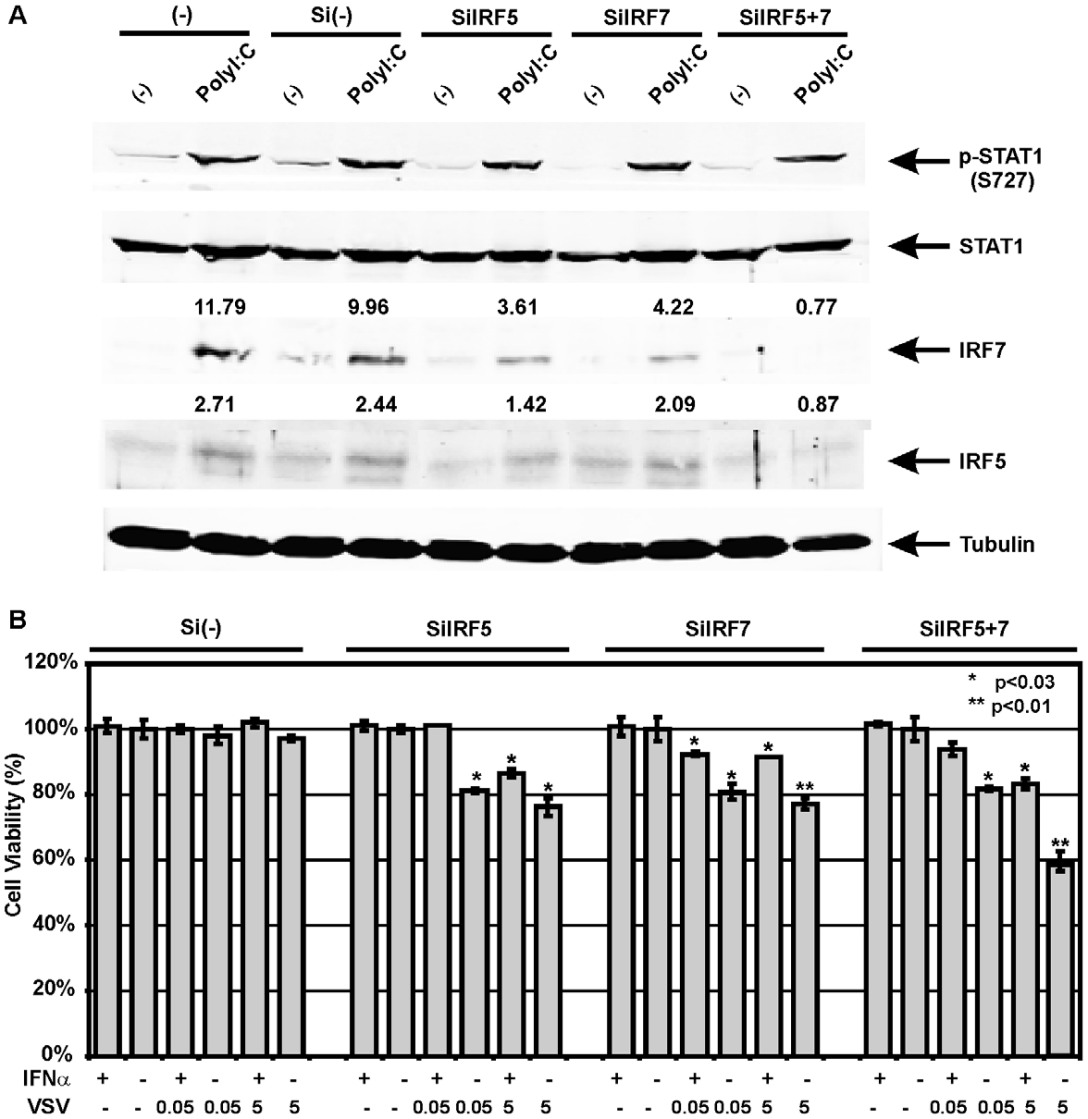
Disruption of IFN pathway increases Beas2B cells' sensitivity to VSV. A. Abrogation of IRF5 and/or IRF7 induction by siRNAs upon polyI:C treatmentwas shown by western blot. Fold changes of IRF5 and IRF7 expression after polyI:C treatment were indicated. Si(-): control siRNA. B. IRF5 and IRF7 are important factors that determine viral sensitivity of the cells. Inhibition of these 2 genes resulted in loss of protection from VSV infection with the most cytotoxicity increase in the combination knockdown. * P<0.03, ** p<0.001.

The selective replication of VSV in tumors with compromised IFN pathway is the cornerstone for its clinical application as oncolytic viral therapy. However, not all lung cancer patients' cancers are deficient in their innate immune pathway. To overcome this obstacle and destroy those VSV-resistant cells, siRNAs to IRF5 (siIRF5) and IRF7 (siIRF7) were applied to suppress IFN response in cells with active IFN pathway. Compared to Beas2B cells transfected with control scrambled siRNA, cells transfected with siIRF5 or siIRF7 resulted in a decrease in IRF5 and IRF7 induction after polyI:C treatment while transfection of both siIRF5+siIRF7 totally eliminated IRF activation (Figure 5A). Transfection of siIRF5 alone significantly inhibited polyI:C-activation of both IRF5 and IRF7, which can be explained by an essential role for IRF5 as a strong inducer of IFNβ [23]. Diminished IRF5 expression by siIRF5 resulted in much less IFNβ production leading to decreased stimulation of secondary response gene IRF7. As expected, the suppression of those 2 genes by siRNAs resulted in significant loss of protection from VSV infection with the most cytotoxicity increase to nearly 40% upon higher dose VSV exposure in the combination knockdowns compared to control siRNA (Figure 5B). Similar findings were observed in parallel transfection studies of VSV-resistant CRL5928 cells, which have relatively intact IFN signaling (data not shown). This clearly demonstrates that IRF5 and IRF7 are functionally essential for innate immunity that determines the viral sensitivity of these cells.

In summary, the loss of IFN signaling is necessary and sufficient for increased sensitivity to killing by oncolytic viruses. The manipulation of the IFN pathway through the transcription factors IRF5 and IRF7 altered the cells' response to VSV infection. Analysis of the IFN pathway using these IRF methylation biomarkers may provide new theranostic biomarkers for determining a patient's sensitivity to oncolytic viruses.

## Discussion

A functional innate immune antiviral system protects cells against viral infection, mainly due to the induction of the defensive IFN signaling cascade, which appears to be the basis for virus resistance and immune stimulatory properties. Here, we have demonstrated the strong and convincing inverse relationship between effective innate antiviral response and oncolytic viral susceptibility using lung cancer cell lines. Moreover, transcription factors IRF5 and IRF7 were identified as critical regulators of innate immune system and useful biomarkers for oncolytic virus susceptibility in lung cancer cells as both of them have to be inducible for a functional IFN pathway. Inactivation of either IRF5 or IRF7 weakened the antiviral response with the most significant loss when both IRFs were epigenetically silenced in CRL5810 cells. The varying extent of cytolytic death was related to the differential severity of IFN pathway defects. Despite the presence of exogenous IFNα, CRL5810 cells are non-responsive and remain vulnerable to VSV killing corresponding to the complete disruption of IFN pathway activity.

Loss of tumor suppressor gene expression by aberrant promoter methylation is an early and common epigenetic event during the onset and progression of cancer [27]. We have shown epigenetic silencing of *IRF5* and *IRF7* at CpG islands of promoter regions in lung cancer. Pharmacological targeting of aberrant epigenetic changes by demethylation agents has shown clinical efficacy in several hematologic malignancies [28]. However, those methylation inhibitors may not prevent the recurrence of hypermethylation and we have presented evidence that epigenetic deregulation cannot be fully reverted in a series of lung cancer cell lines. The failure of demethylation agents to restore crucial ISG expression indicates that those lung cancer cells are ideal targets for oncolytic viral therapy.

Although basal levels of most ISGs were reduced in all the lung cancer cell lines compared to normal bronchial epithelial cells, oncolytic viral sensitivity is only associated with polyI:C-inducible expression levels of certain key ISGs such as IRF7 and IRF5. However, IRF5 and IRF7 overexpression is not sufficient to completely restore IFN pathway and only partially rescued cells from viral infection due to deficiency of other important ISGs. Failure to induce Ser727 phosphorylation upon polyI:C treatment suggested additional innate immunity defects in several VSV sensitive cell lines (Figure 1B). In addition, mRNA of 3 other ISGs (STAT1, IRF3 and IFI16, Table 2) is consistently induced in VSV-resistant cells. Further studies are needed to confirm them as theranostic biomarkers and may identify additional ISGs as biomarkers in lung cancer whose activation could distinguish virus-resistant from virus-sensitive cancers.

We presented evidence that functional inactivation of IRF5 and IRF7 is the major mechanism to disrupt IFN signaling in lung cancer cells. Nevertheless, various malignancies harbor diverse molecular defects of this pathway. Deregulated JAK3 and RNase L pathways in LNCaP prostate cancer cells [29], defective STAT1 and STAT2 activation in fibrosarcoma and melanoma cells [30], [31] and down-regulated IFNAR in high grade bladder cancer [32] have all been reported to disable the innate immune signaling. Overall, the IFN pathway is frequently downregulated during tumorigenesis even though distinct sets of ISGs are suppressed by different mechanisms in a cancer-type-specific manner.

A failure to activate innate immunity response upon oncolytic RNA virus infection leads to the highly selective clearance of IFN-nonresponsive tumor cells. In addition to VSV, other RNA viruses such as NDV [33] and influenza virus [34] have also been demonstrated to have tumor-selective cytotoxicity using the same mechanism to target cells with diminished IFN activity. Clinical trials of intravenous administration of NDV (PV701) proved its initial oncolytic effects in several solid tumors [35]. In addition, VSV strains with M protein mutations (AV1 and AV2) triggered more robust antiviral response because of their defects in the ability to shutdown IFN signaling; these mutants are selectively destroyed in IFN-responsive cells at a lower therapeutic dose while remaining highly lytic in cancer cells [9]. Expression of immune stimulating proteins such as interleukin-2 (IL-4) or IFNβ in genetically engineered VSV has also been generated to promote viral cytolytic responses [5], [6]. Hence, boosting anti-viral responses in normal cells will enhance oncolytic selectivity in IFN-nonresponsive tumors.

Although a reduced antiviral response may be a common feature of a broad range of cancers, the oncolytic efficacy of naturally occurring RNA viruses may still be relatively low, as some tumors manifest robust innate immunity responses that inhibit viral replication and spread. To overcome this obstacle, damping of cellular IFN responses in cancer cells by IFN-antagonist, such as influenza NS1 or inhibition of IFN-stimulating kinase (mTORC1) have been demonstrated to be effective strategies to augment therapeutic viral activity [36], [37]. Another study showed that down-regulation of IFNAR1 sensitized bladder cancer cells to VSV-induced cell death [32]. In this report we eliminated IFN signaling using specific siRNAs to essential IRFs, which potentiated cytolysis killing by VSV. Therefore small molecule manipulation of the innate immune response could, in the future, modulate the cellular response to oncolytic RNA viruses to make them more effective.

The abrogated IFN response in more than 80% of human cancers favors the selective viral replication and cytotoxicity in those tumor cells and makes them ideal targets for oncolytic virus treatment [9]. Our results support the use of a diminished innate immune response due to epigenetic silencing by promoter methylation of *IRF5* and *IRF7* as a theranostic strategy for oncolytic virus VSV treatment of lung tumors. Because not all of the lung cancer patients may benefit from VSV oncolytic viral therapy due to relatively normal function of the IFN pathway in some cancer cells, using IRF-promoter methylation as theranostic method for developing ISG-promoter related biomarkers is capable of assessing the susceptibility of each specific cancer case. Its absence limits the successful application of oncolytic therapy, causing delay of other more effective treatment and unnecessary side effects in VSV resistant patients [38]. Screening individual patients using those ISG biomarkers is necessary as only those individuals with defective innate immune system will benefit from VSV treatment. These ISG biomarkers will not only determine cells' innate immunity status and sensitivity to oncolytic viruses such as VSV but also provide future possibilities for IFN pathway manipulation to make resistant tumors more vulnerable to oncolytic virus killing by targeting these key ISGs.

## Materials and Methods

### Cell culture

Beas2B cell line was derived from immortalization of normal human bronchial epithelial cells (NHBE) with SV40-adenovirus E1a hybrid virus. Beas2B cells were used as normal control cells as they retain many properties of NHBE including potential for terminal differentiation; they are believed to represent the normal progenitor cells for lung carcinomas [39]. Beas2B cells were grown with LHC-9 media (Invitrogen, Carlsbad, CA) in a 37°C, 5% CO2 incubator in dishes precoated with fibronectin (BD Biosciences, San Jose, CA), type I collagen and Bovine Albumin Fraction V (Invitrogen, Carlsbad, CA). Lung cancer cell lines CRL5928 and CRL5869 were obtained from ATCC, Manassas, VA and the remaining cell lines were kind gifts of Dr. Anil Wali. All the lung cancer cell lines were cultured in RMPI1640 media with 10% fetal bovine serum (Invitrogen, Carlsbad, CA)) in a 37°C, 5% CO2 incubator.

### Primary lung cancer tissue collection

Twenty fresh frozen primary non-small-cell lung carcinoma (NSCLC) tissue samples and buffy coats from the same lung cancer patients were obtained from The Ontario Tumour Bank, Toronto, Ontario, Canada. This included 12 adenocarcinomas and 8 squamous cell carcinoma tissues. Detailed information of those samples is listed in Table S1.

### 5-aza-dC and polyI:C treatment

5-aza-dC (Sigma-Aldrich, Inc., Sainte Louis, MO) treatment was done followed the protocol described earlier [14]. Polyinosinic:polycytidylic acid (polyI:C) (Amersham Biosciences Corp., Piscataway, NJ) was diluted according to manufacture's instruction and 100 µ/ml polyI:C was applied on Beas2B and lung cancer cells for 24 hours. The untreated control cells were changed with fresh media for the same period of time before total RNA and protein were harvested.

### Quantitative RT-PCR

Total RNA was extracted from each experiment using the QIAGEN RNeasy Kit (QIAGEN, Inc., Valencia, CA). Two µg total RNA was reverse transcribed into cDNA using Superscript II (Invitrogen, Carlsbad, CA). Q-RT-PCR was performed using SYBR Green PCR Detection kit (PE Biosystems, Warrington, U.K.) as described previously [14]. The primer sets used are listed in Table S2. The housekeeping gene GAPDH was used as a normalizing control.

### Western blots

Western blots were performed as described [15] using 50 µg of cell extracts. Primary antibodies used in our study were rabbit anti-IRF5 and chicken anti-OAS1 (Abcam Inc., Cambridge, MA); rabbit anti-STAT1, rabbit anti-IRF7 and mouse anti-α-tubulin (Santa Cruz Biotechnology, Inc. Santa Cruz, CA); rabbit antiphospho-STAT1 (Ser727) (Cell Signaling Technology, Inc., Beverly, MA) and mouse anti-flag (Sigma-Aldrich, Inc., Sainte Louis, MO).

### PCR amplification, cloning and sequencing of bisulfite modified genomic DNA

Genomic DNA for lung cancer cell lines, primary lung cancer patient tissues, and buffy coat DNAs from those patients were prepared using QIAamp DNA kit (QIAGEN, Inc., Valencia, CA). Genomic DNA (0.5 µg) was denatured and bisulfite converted using EZ DNA methylation-GOLD kit (Zymo Research, Orange, CA). The bisulfite modified genomic DNA was suspended in 10 µl of water and 2 µl of DNA was amplified by PCR with two nested PCR reactions. The annealing temperature was 56°C for the first PCR and 58°C for the second PCR. The two sets of primers for IRF7 are:

F1 5′ GTAAGGGTTTTTGTCGTAGTAGACGTTAG;
R1 5′ AACGTAATAATTCATACCTATAATCCCAAC;
F2 5′ GGTTATAGGTGTGATTGTAGGTGTG;
R2 5′ CCCTAAACTATAATAAAATAACTCCATCTC.

The two sets of primers for IRF5 are:

F1 5′ TGATTGGAAGGCGATTTAGG;
R1 5′ AAAATCCCAAACCGACCGAA;
F2 5′ AGTGGGGAAGTATTTTATTTTTTTT;
R2 5′ CCCCTAAACAACTACTACTAAACTCC.

The PCR products were subjected to double-strand DNA sequencing using primer F2.

### VSV sensitivity assay

Cells were seeded in 96 well plates at a density of 1−2×10^4^ cells per well and cultured overnight in the presence or absence of IFNα (Biosource International, Inc., Camarillo, CA. 1000 U/ml). The cells were then infected with a low dose (multiplicity of infection, MOI = 0.05) or high dose (MOI = 5.0) of VSV (Indiana strain) for 1 hr. Virus-induced cytopathicity was determined the next day by modified version of MTT assay as described previously [15]. Results were expressed as relative values of cell viability compared to control uninfected cells (set to 100).

### Manipulation of IFN pathway by IRF overexpression or siRNA disruption

pCMV-IRF7, pCMV-IRF5 and control vector pcDNA3.1 were stably transfected into CRL5810 cells followed by 200 µg/ml G418 selection as described previously [15]. SiRNAs targeting IRF5 and IRF7 and control siRNA were transfected into Beas2B and CRL5928 cells using siRNA transfection reagent (all from Santa Cruz Biotechnology, Inc. Santa Cruz, CA). Forty-eight hours later, the siRNA transfected cells were treated with 100 µg/ml polyI:C for an additional 24 hours before western blots were used to examine protein expressions of several ISGs. VSV sensitivity assays were performed on both IRF-overexpressed and siRNA- transfected cells.

## Supporting Information

Table S1 **Information about collected lung cancer tissue samples.**
(DOC)

Table S2 **List of primer sets used in Q-RT-PCR.**
(DOC)

## References

[1] Wong HH, Lemoine NR, Wang Y (2010). Oncolytic Viruses for Cancer Therapy: Overcoming the Obstacles.. Viruses.

[2] Naik S, Russell SJ (2009). Engineering oncolytic viruses to exploit tumor specific defects in innate immune signaling pathways.. Expert Opin Biol Ther.

[3] Rowan K (2010). Oncolytic viruses move forward in clinical trials.. J Natl Cancer Inst.

[4] Balachandran S, Porosnicu M, Barber GN (2001). Oncolytic activity of vesicular stomatitis virus is effective against tumors exhibiting aberrant p53, Ras, or myc function and involves the induction of apoptosis.. J Virol.

[5] Fernandez M, Porosnicu M, Markovic D, Barber GN (2002). Genetically engineered vesicular stomatitis virus in gene therapy: application for treatment of malignant disease.. J Virol.

[6] Obuchi M, Fernandez M, Barber GN (2003). Development of recombinant vesicular stomatitis viruses that exploit defects in host defense to augment specific oncolytic activity.. J Virol.

[7] Wollmann G, Robek MD, van den Pol AN (2007). Variable deficiencies in the interferon response enhance susceptibility to vesicular stomatitis virus oncolytic actions in glioblastoma cells but not in normal human glial cells.. J Virol.

[8] Stojdl DF, Lichty B, Knowles S, Marius R, Atkins H (2000). Exploiting tumor-specific defects in the interferon pathway with a previously unknown oncolytic virus.. Nat Med.

[9] Stojdl DF, Lichty BD, tenOever BR, Paterson JM, Power AT (2003). VSV strains with defects in their ability to shutdown innate immunity are potent systemic anti-cancer agents.. Cancer Cell.

[10] Dunn GP, Koebel CM, Schreiber RD (2006). Interferons, immunity and cancer immunoediting.. Nat Rev Immunol.

[11] Picaud S, Bardot B, De Maeyer E, Seif I (2002). Enhanced tumor development in mice lacking a functional type I interferon receptor.. J Interferon Cytokine Res.

[12] Uno K, Hirosaki M, Kakimi K, Tominaga M, Suginoshita Y (2007). Impaired IFN-alpha production and the risk of cancer development.. J Interferon Cytokine Res.

[13] Fridman AL, Tang L, Kulaeva OI, Ye B, Li Q (2006). Expression profiling identifies three pathways altered in cellular immortalization: interferon, cell cycle, and cytoskeleton.. J Gerontol A Biol Sci Med Sci.

[14] Kulaeva OI, Draghici S, Tang L, Kraniak JM, Land SJ (2003). Epigenetic silencing of multiple interferon pathway genes after cellular immortalization.. Oncogene.

[15] Li Q, Tang L, Roberts PC, Kraniak JM, Fridman AL (2008). Interferon regulatory factors IRF5 and IRF7 inhibit growth and induce senescence in immortal Li-Fraumeni fibroblasts.. Mol Cancer Res.

[16] Fukasawa M, Kimura M, Morita S, Matsubara K, Yamanaka S (2006). Microarray analysis of promoter methylation in lung cancers.. J Hum Genet.

[17] Jee CD, Kim MA, Jung EJ, Kim J, Kim WH (2009). Identification of genes epigenetically silenced by CpG methylation in human gastric carcinoma.. Eur J Cancer.

[18] Kumagai T, Akagi T, Desmond JC, Kawamata N, Gery S (2009). Epigenetic regulation and molecular characterization of C/EBPalpha in pancreatic cancer cells.. Int J Cancer.

[19] Yu J, Zhang HY, Ma ZZ, Lu W, Wang YF (2003). Methylation profiling of twenty four genes and the concordant methylation behaviours of nineteen genes that may contribute to hepatocellular carcinogenesis.. Cell Res.

[20] Barnes BJ, Kellum MJ, Pinder KE, Frisancho JA, Pitha PM (2003). Interferon regulatory factor 5, a novel mediator of cell cycle arrest and cell death.. Cancer Res.

[21] Shin SH, Kim BH, Jang JJ, Suh KS, Kang GH (2010). Identification of novel methylation markers in hepatocellular carcinoma using a methylation array.. J Korean Med Sci.

[22] Yamashita M, Toyota M, Suzuki H, Nojima M, Yamamoto E (2010). DNA methylation of interferon regulatory factors in gastric cancer and noncancerous gastric mucosae.. Cancer Sci.

[23] Barnes BJ, Richards J, Mancl M, Hanash S, Beretta L (2004). Global and distinct targets of IRF-5 and IRF-7 during innate response to viral infection.. J Biol Chem.

[24] Sanceau J, Hiscott J, Delattre O, Wietzerbin J (2000). IFN-beta induces serine phosphorylation of Stat-1 in Ewing's sarcoma cells and mediates apoptosis via induction of IRF-1 and activation of caspase-7.. Oncogene.

[25] Jaspers I, Horvath KM, Zhang W, Brighton LE, Carson JL (2010). Reduced expression of IRF7 in nasal epithelial cells from smokers after infection with influenza.. Am J Respir Cell Mol Biol.

[26] Singal R, vanWert JM (2001). De novo methylation of an embryonic globin gene during normal development is strand specific and spreads from the proximal transcribed region.. Blood.

[27] Ellis L, Atadja PW, Johnstone RW (2009). Epigenetics in cancer: targeting chromatin modifications.. Mol Cancer Ther.

[28] Issa JP (2007). DNA methylation as a therapeutic target in cancer.. Clin Cancer Res.

[29] Dong B, Kim S, Hong S, Das Gupta J, Malathi K (2007). An infectious retrovirus susceptible to an IFN antiviral pathway from human prostate tumors.. Proc Natl Acad Sci U S A.

[30] Krishnamurthy S, Takimoto T, Scroggs RA, Portner A (2006). Differentially regulated interferon response determines the outcome of Newcastle disease virus infection in normal and tumor cell lines.. J Virol.

[31] Wong LH, Krauer KG, Hatzinisiriou I, Estcourt MJ, Hersey P (1997). Interferon-resistant human melanoma cells are deficient in ISGF3 components, STAT1, STAT2, and p48-ISGF3gamma.. J Biol Chem.

[32] Zhang KX, Matsui Y, Hadaschik BA, Lee C, Jia W (2010). Down-regulation of type I interferon receptor sensitizes bladder cancer cells to vesicular stomatitis virus-induced cell death.. Int J Cancer.

[33] Wilden H, Fournier P, Zawatzky R, Schirrmacher V (2009). Expression of RIG-I, IRF3, IFN-beta and IRF7 determines resistance or susceptibility of cells to infection by Newcastle Disease Virus.. Int J Oncol.

[34] Muster T, Rajtarova J, Sachet M, Unger H, Fleischhacker R (2004). Interferon resistance promotes oncolysis by influenza virus NS1-deletion mutants.. Int J Cancer.

[35] Pecora AL, Rizvi N, Cohen GI, Meropol NJ, Sterman D (2002). Phase I trial of intravenous administration of PV701, an oncolytic virus, in patients with advanced solid cancers.. J Clin Oncol.

[36] Alain T, Lun X, Martineau Y, Sean P, Pulendran B (2010). Vesicular stomatitis virus oncolysis is potentiated by impairing mTORC1-dependent type I IFN production.. Proc Natl Acad Sci U S A.

[37] Zamarin D, Martinez-Sobrido L, Kelly K, Mansour M, Sheng G (2009). Enhancement of oncolytic properties of recombinant newcastle disease virus through antagonism of cellular innate immune responses.. Mol Ther.

[38] Hotte SJ, Lorence RM, Hirte HW, Polawski SR, Bamat MK (2007). An optimized clinical regimen for the oncolytic virus PV701.. Clin Cancer Res.

[39] Ke Y, Reddel RR, Gerwin BI, Miyashita M, McMenamin M (1988). Human bronchial epithelial cells with integrated SV40 virus T antigen genes retain the ability to undergo squamous differentiation.. Differentiation.

