# Supplementary material for: Epigenetic Silencing of IRF7 and/or IRF5 in Lung Cancer Cells Leads to Increased Sensitivity to Oncolytic Viruses
Source: PLoS One. 2011 Dec 14;6(12):e28683. doi: 10.1371/journal.pone.0028683 (PMC3237484; doi:10.1371/journal.pone.0028683)
Supplement: Table S1 — Information about collected lung cancer tissue samples. (DOC) [file pone.0028683.s001.doc]

Table S1. Information about collected lung cancer tissue samples.

| **DONOR** | **SEX** | **AGE** | **HISTOLOGY DESCRIPTION** | **GRADE** | **STAGE** |
| --- | --- | --- | --- | --- | --- |
| A00167 | F | 64 | Adenocarcinoma | IIIA | T3N2M0 |
| A00316 | M | 75 | Adenocarcinoma | II | T2N0MX |
| A00409 | F | 59 | Adenocarcinoma | IB | T2N0MX |
| A01013 | F | 63 | Adenocarcinoma | IIIB | T2N0M0 |
| A01173 | F | 68 | Adenocarcinoma | IB | T2N0MX |
| B00159 | M | 71 | Adenocarcinoma | IIIA | T2N2M0 |
| B00175 | F | 54 | Adenocarcinoma | IV | T2N1M1 |
| B00915 | F | 67 | Adenocarcinoma | IV | T2N1M1 |
| B01430 | M | 72 | Adenocarcinoma | IB | T2N0M0 |
| D01300 | M | 78 | Adenocarcinoma | IIIB | T4N0M0 |
| D01562 | F | 59 | Adenocarcinoma | II | T1N1M0 |
| D02069 | M | 72 | Adenocarcinoma | IIB | T2N1M0 |
| A01353 | M | 57 | Squamous carcinoma | IIB | T3N0M0 |
| B00196 | M | 73 | Squamous carcinoma | IIB | T3N0M0 |
| B00711 | F | 69 | Squamous carcinoma | IIIA | T3N1M0 |
| B01060 | M | 79 | Squamous carcinoma | IIB | T2N1M0 |
| B01740 | M | 48 | Squamous carcinoma | III | T2N0M0 |
| B02305 | F | 76 | Squamous carcinoma | II | T2N0M0 |
| D02120 | M | 66 | Squamous carcinoma | IV | T3N2M1 |
| B02420 | F | 82 | Squamous carcinoma | II | T2N0M0 |
