# Supplementary material for: Epigenetic Silencing of IRF7 and/or IRF5 in Lung Cancer Cells Leads to Increased Sensitivity to Oncolytic Viruses
Source: PLoS One. 2011 Dec 14;6(12):e28683. doi: 10.1371/journal.pone.0028683 (PMC3237484; doi:10.1371/journal.pone.0028683)
Supplement: Table S2 — List of primer sets used in Q-RT-PCR. (DOC) [file pone.0028683.s002.doc]

Table S2. List of primer sets used in Q-RT-PCR.

| Gene | Forward primer1 | Reverse primer1 |
| --- | --- | --- |
| *GAPDH* | ATCAAGAAGGTGGTGAAGCAG | TGTCGCTGTTGAAGTCAGAGG |
| *TLR3* | ATCCCTGAGCTGTCAAGCCA | GGGCACTGTCTTTGCAAGATG |
| *IFNa* | CAGTTCCAGAAGGCTCCAGC | TCCTCATCCCAAGCAGCAG |
| *IFNb* | GGCAATTGAATGGAGGCT | GGCGTCCTCCTTCTGGAACT |
| *IFN-regulatory factor 7, IRF7* | GCAGCGTGAGGGTGTGTCTT | GCTCCATAAGGAAGCACTCGAT |
| *IFN-regulatory factor 5, IRF5* | TTCTCTCCTGGGCTGTCTCTG | CTATACAGCTAGGCCCCAGGG |
| *STAT1a* | CTGCTGCGGTTCAGTGAGAG | GGTTCAACCGCATGGAAGTC |
| *OAS1* | TGAGGCCTGGCTGAATTACC | TGAATGGCAGGGAGGAAGC |
| *IFN-induced 17-kDa/15-kDa protein, IFI15* | CAAATGCGACGAACCTCTGAG | GCTGCTTCAGGTGGGCC |
| *IFN-inducible protein p78, Mx1* | GCCACTGGACTGACGACTTGA | ACTGCTCTCACAGCTTCCTGC |
| *Type I IFN Receptor 1, IFNAR1* | CAAGCTTTCCTACTTCCTCCA | CACAGGCGTGTTTCCAGAC |
| *IFN-regulatory factor 3, IRF3* | CAGCTTGGACAATCCCACTC | GTCACCTCGAACTCCCACTC |
| *IFN-induced 16-kDa protein, IFI16* | GTCCGAGGAACAGACTCAGC | GGTGGAGCTGACAATGAGGT |
| *IFN-inducible protein p27, IFI27* | TCCTCCATAGCAGCCAAGATG | GTTGCTCCCAGTGACTGCAG |
| ^1^All primers have a 5’-3’ orientation |  |  |
